# STrengthening the REporting of Genetic Association Studies (STREGA)— An Extension of the STROBE Statement

**DOI:** 10.1371/journal.pmed.1000022

**Published:** 2009-02-03

**Authors:** Julian Little, Julian P.T Higgins, John P.A Ioannidis, David Moher, France Gagnon, Erik von Elm, Muin J Khoury, Barbara Cohen, George Davey-Smith, Jeremy Grimshaw, Paul Scheet, Marta Gwinn, Robin E Williamson, Guang Yong Zou, Kim Hutchings, Candice Y Johnson, Valerie Tait, Miriam Wiens, Jean Golding, Cornelia van Duijn, John McLaughlin, Andrew Paterson, George Wells, Isabel Fortier, Matthew Freedman, Maja Zecevic, Richard King, Claire Infante-Rivard, Alex Stewart, Nick Birkett

**Affiliations:** 1 Canada Research Chair in Human Genome Epidemiology; 2 Department of Epidemiology and Community Medicine, University of Ottawa, Ottawa, Ontario, Canada; 3 MRC Biostatistics Unit, Cambridge, United Kingdom; 4 Department of Hygiene and Epidemiology, School of Medicine, University of Ioannina, Ioannina, Greece; and Center for Genetic Epidemiology and Modeling, Tufts University School of Medicine, Boston, Massachusetts, United States of America; 5 CIHR New Investigator and Canada Research Chair in Genetic Epidemiology, University of Toronto, Dalla Lana School of Public Health, Toronto, Ontario, Canada; 6 Institute of Social and Preventive Medicine, University of Bern, Bern, Switzerland; and German Cochrane Centre, Department of Medical Biometry and Medical Informatics, University Medical Centre, Freiburg, Germany; 7 National Office of Public Health Genomics, Centers for Disease Control and Prevention, Atlanta, Georgia, United States of America; 8 former Senior Editor, Public Library of Science, San Francisco, California, United States of America; 9 MRC Centre for Causal Analyses in Translational Epidemiology, Department of Social Medicine, University of Bristol, Bristol, United Kingdom; 10 Canada Research Chair in Health Knowledge Transfer and Uptake, Clinical Epidemiology Program, Ottawa Health Research Institute, Department of Medicine, University of Ottawa, Ottawa, Ontario, Canada; 11 University of Texas, MD Anderson Cancer Center, Department of Epidemiology, Houston, Texas, United States of America; 12 Deputy Editor, *American Journal of Human Genetics*, Boston, Massachusetts, United States of America; 13 Department of Epidemiology and Biostatistics, University of Western Ontario, London, Ontario, Canada; and Robarts Clinical Trials, Robarts Research Institute, London, Ontario, Canada; 14 Editor, *Paediatric and Perinatal Epidemiology*, Bristol, United Kingdom; 15 Editor, *European Journal of Epidemiology*, Rotterdam, The Netherlands; 16 Cancer Care Ontario, Toronto, Ontario, Canada; and Prosserman Centre for Health Research at the Samuel Lunenfeld Research Institute, Toronto, Ontario, Canada; 17 Canada Research Chair in Genetics of Complex Diseases, Hospital for Sick Children (SickKids), Toronto, Ontario, Canada; 18 Director, Cardiovascular Research Methods Centre, University of Ottawa Heart Institute, Ottawa, Ontario, Canada; 19 Genome Quebec & P3G Observatory, McGill University and Genome Quebec Innovation Center, Montreal, Quebec, Canada; 20 Dana-Farber Cancer Institute, Boston, Massachusetts, United States of America; 21 Senior Editor, *Lancet*, New York, New York, United States of America; 22 former Editor, *Genetics in Medicine*, Minneapolis, Minnesota, United States of America; 23 Canada Research Chair-James McGill Professor, Department of Epidemiology, Biostatistics and Occupational Health, Faculty of Medicine, McGill University, Montreal, Quebec, Canada; 24 University of Ottawa Heart Institute, Ottawa, Ontario, Canada

**Keywords:** gene-disease associations, genetics, gene-environment interaction, systematic review, meta analysis, reporting recommendations, epidemiology, genome-wide association

## Abstract

Julian Little and colleagues present the STREGA recommendations, which are aimed at improving the reporting of genetic association studies.

The rapidly evolving evidence on genetic associations is crucial to integrating human genomics into the practice of medicine and public health [1,2]. Genetic factors are likely to affect the occurrence of numerous common diseases, and therefore identifying and characterizing the associated risk (or protection) will be important in improving the understanding of etiology and potentially for developing interventions based on genetic information. The number of publications on the associations between genes and diseases has increased tremendously; with more than 34 000 published articles, the annual number has more than doubled between 2001 and 2008 [3,4]. Articles on genetic associations have been published in about 1500 journals and in several languages.

Despite the many similarities between genetic association studies and “classical” observational epidemiologic studies (that is, cross-sectional, case-control, and cohort) of lifestyle and environmental factors, genetic association studies present several specific challenges including an unprecedented volume of new data [5,6] and the likelihood of very small individual effects. Genes may operate in complex pathways with gene-environment and gene-gene interactions [7]. Moreover, the current evidence base on gene-disease associations is fraught with methodological problems [8–10]. Inadequate reporting of results, even from well-conducted studies, hampers assessment of a study's strengths and weaknesses, and hence the integration of evidence [11].

**Summary**

Making sense of rapidly evolving evidence on genetic associations is crucial to making genuine advances in human genomics and the eventual integration of this information in the practice of medicine and public health. Assessment of the strengths and weaknesses of this evidence, and hence the ability to synthesize it, has been limited by inadequate reporting of results. The STrengthening the REporting of Genetic Association studies (STREGA) initiative builds on the Strengthening the Reporting of Observational Studies in Epidemiology (STROBE) Statement and provides additions to 12 of the 22 items on the STROBE checklist. The additions concern population stratification, genotyping errors, modelling haplotype variation, Hardy-Weinberg equilibrium, replication, selection of participants, rationale for choice of genes and variants, treatment effects in studying quantitative traits, statistical methods, relatedness, reporting of descriptive and outcome data, and the volume of data issues that are important to consider in genetic association studies. The STREGA recommendations do not prescribe or dictate how a genetic association study should be designed but seek to enhance the transparency of its reporting, regardless of choices made during design, conduct, or analysis.

Although several commentaries on the conduct, appraisal and/or reporting of genetic association studies have so far been published [12–39], their recommendations differ. For example, some papers suggest that replication of findings should be part of the publication [12,13,16,17,23,26,34–36] whereas others consider this suggestion unnecessary or even unreasonable [21,40–44]. In many publications, the guidance has focused on genetic association studies of specific diseases [14,15,17,19,22,23,25,26,31–38] or the design and conduct of genetic association studies [13–15,17,19,20,22,23,25,30–32,35,36] rather than on the quality of the reporting.

Despite increasing recognition of these problems, the quality of reporting genetic association studies needs to be improved [45–49]. For example, an assessment of a random sample of 315 genetic association studies published from 2001 to 2003 found that most studies provided some qualitative descriptions of the study participants (for example, origin and enrolment criteria), but reporting of quantitative descriptors, such as age and sex, was variable [49]. In addition, completeness of reporting of methods that allow readers to assess potential biases (for example, number of exclusions or number of samples that could not be genotyped) varied [49]. Only some studies described methods to validate genotyping or mentioned whether research staff were blinded to outcome. The same problems persisted in a smaller sample of studies published in 2006 [49]. Lack of transparency and incomplete reporting have raised concerns in a range of health research fields [11,50–53] and poor reporting has been associated with biased estimates of effects in clinical intervention studies [54].

The main goal of this article is to propose and justify a set of guiding principles for reporting results of genetic association studies. The epidemiology community has recently developed the Strengthening the Reporting of Observational studies in Epidemiology (STROBE) Statement for cross-sectional, case-control, and cohort studies [55,56]. Given the relevance of general epidemiologic principles for genetic association studies, we propose recommendations in an extension of the STROBE Statement called the STrengthening the REporting of Genetic Association studies (STREGA) Statement. The recommendations of the STROBE Statement have a strong foundation because they are based on empirical evidence on the reporting of observational studies, and they involved extensive consultations in the epidemiologic research community [56]. We have sought to identify gaps and areas of controversy in the evidence regarding potential biases in genetic association studies. With the recommendations, we have indicated available empirical or theoretical work that has demonstrated or suggested that a methodological feature of a study can influence the direction or magnitude of the association observed. We acknowledge that for many items, no such evidence exists. The intended audience for the reporting guideline is broad and includes epidemiologists, geneticists, statisticians, clinician scientists, and laboratory-based investigators who undertake genetic association studies. In addition, it includes “users” of such studies who wish to understand the basic premise, design, and limitations of genetic association studies in order to interpret the results. The field of genetic associations is evolving very rapidly with the advent of genome-wide association investigations, high-throughput platforms assessing genetic variability beyond common single nucleotide polymorphisms (SNPs) (for example, copy number variants, rare variants), and eventually routine full sequencing of samples from large populations. Our recommendations are not intended to support or oppose the choice of any particular study design or method. Instead, they are intended to maximize the transparency, quality and completeness of reporting of what was done and found in a particular study.

## Methods

A multidisciplinary group developed the STREGA Statement by using literature review, workshop presentations and discussion, and iterative electronic correspondence after the workshop. Thirty-three of 74 invitees participated in the STREGA workshop in Ottawa, Ontario, Canada, in June, 2006. Participants included epidemiologists, geneticists, statisticians, journal editors and graduate students.

Before the workshop, an electronic search was performed to identify existing reporting guidance for genetic association studies. Workshop participants were also asked to identify any additional guidance. They prepared brief presentations on existing reporting guidelines, empirical evidence on reporting of genetic association studies, the development of the STROBE Statement, and several key areas for discussion that were identified on the basis of consultations before the workshop. These areas included the selection and participation of study participants, rationale for choice of genes and variants investigated, genotyping errors, methods for inferring haplotypes, population stratification, assessment of Hardy-Weinberg equilibrium (HWE), multiple testing, reporting of quantitative (continuous) outcomes, selectively reporting study results, joint effects and inference of causation in single studies. Additional resources to inform workshop participants were the HuGENet handbook [57,58], examples of data extraction forms from systematic reviews or meta-analyses, articles on guideline development [59,60] and the checklists developed for STROBE. To harmonize our recommendations for genetic association studies with those for observational epidemiologic studies, we communicated with the STROBE group during the development process and sought their comments on the STREGA draft documents. We also provided comments on the developing STROBE Statement and its associated explanation and elaboration document [56].

## Results

In Table 1, we present the STREGA recommendations, an extension to the STROBE checklist [55] for genetic association studies (an editable version of Table 1 is provided as Table S1 under Supporting Information). The resulting STREGA checklist provides additions to 12 of the 22 items on the STROBE checklist. During the workshop and subsequent consultations, we identified five main areas of special interest that are specific to, or especially relevant in, genetic association studies: genotyping errors, population stratification, modelling haplotype variation, HWE and replication. We elaborate on each of these areas, starting each section with the corresponding STREGA recommendation, followed by a brief outline of the issue and an explanation for the recommendations. Complementary information on these areas and the rationale for additional STREGA recommendations relating to selection of participants, choice of genes and variants selected, treatment effects in studying quantitative traits, statistical methods, relatedness, reporting of descriptive and outcome data, and issues of data volume, are presented in Table 2.

**Table 1:**
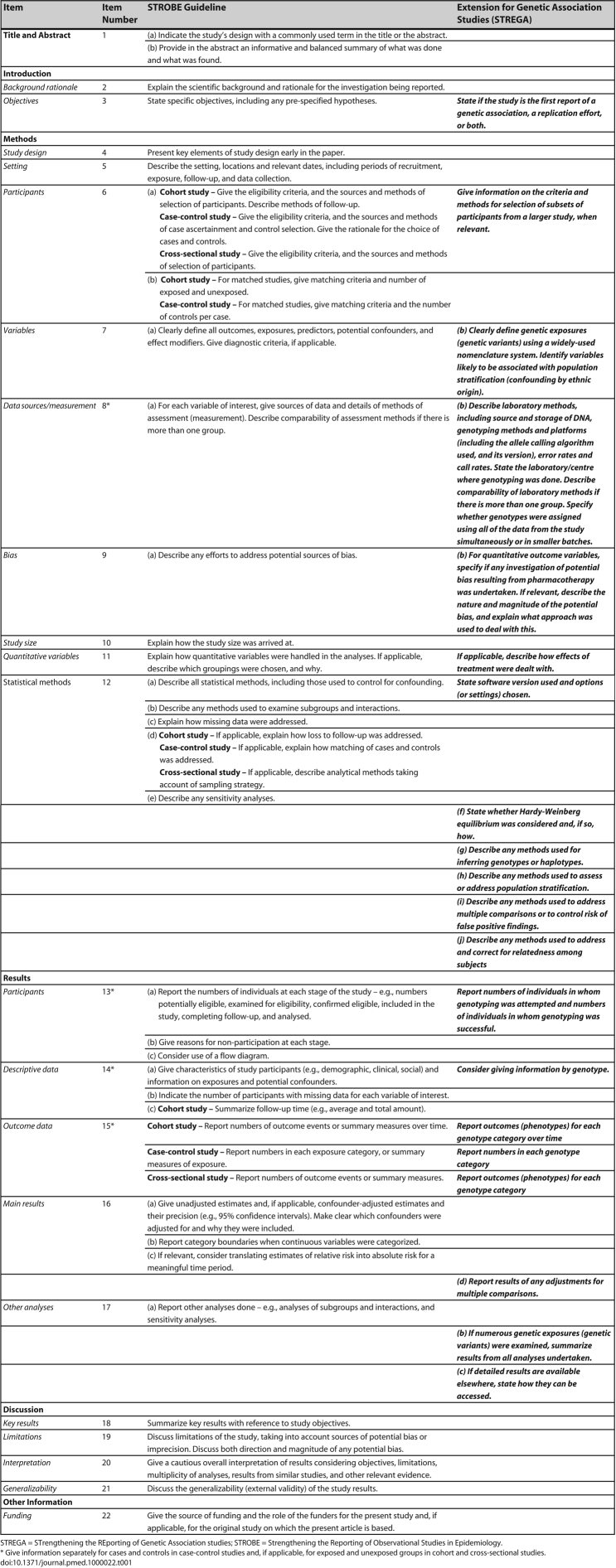
STREGA Reporting Recommendations, Extended from STROBE Statement

**Table 2:**
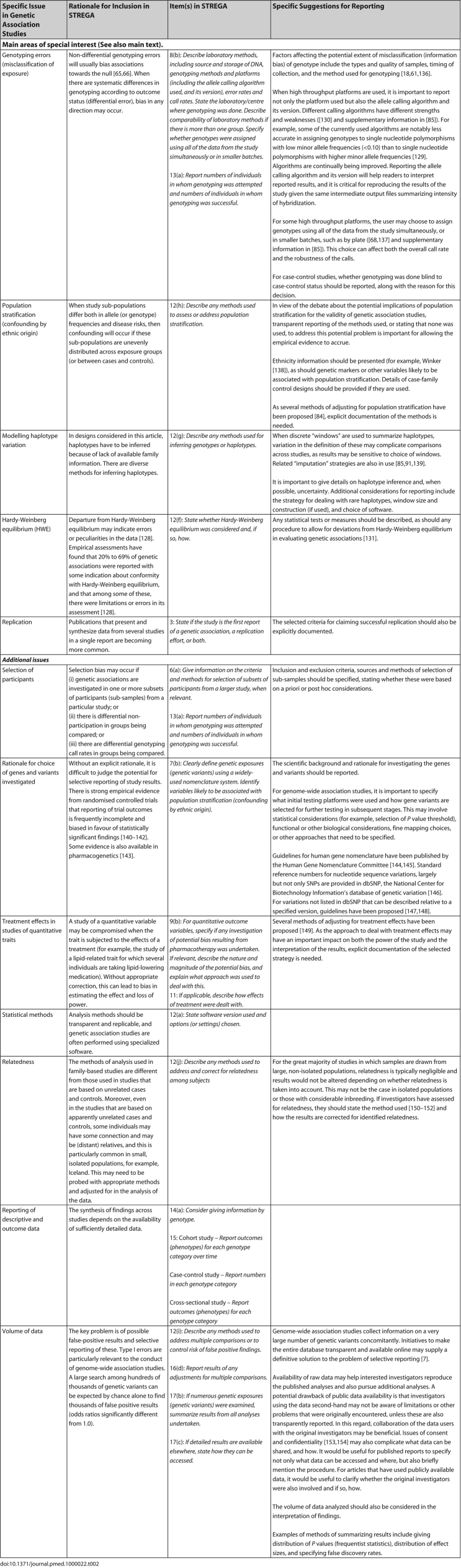
Rationale for Inclusion of Topics in the STREGA Recommendations

### Genotyping Errors

*Recommendation for reporting of methods (Table 1, item 8(b)): Describe laboratory methods, including source and storage of DNA, genotyping methods and platforms (including the allele calling algorithm used, and its version), error rates and call rates. State the laboratory/centre where genotyping was done. Describe comparability of laboratory methods if there is more than one group. Specify whether genotypes were assigned using all of the data from the study simultaneously or in smaller batches.*

*Recommendation for reporting of results (Table 1, item 13(a)): Report numbers of individuals in whom genotyping was attempted and numbers of individuals in whom genotyping was successful.*

Genotyping errors can occur as a result of effects of the DNA sequence flanking the marker of interest, poor quality or quantity of the DNA extracted from biological samples, biochemical artefacts, poor equipment precision or equipment failure, or human error in sample handling, conduct of the array or handling the data obtained from the array [61]. A commentary published in 2005 on the possible causes and consequences of genotyping errors observed that an increasing number of researchers were aware of the problem, but that the effects of such errors had largely been neglected [61]. The magnitude of genotyping errors has been reported to vary between 0.5% and 30% [61–64]. In high-throughput centres, an error rate of 0.5% per genotype has been observed for blind duplicates that were run on the same gel [64]. This lower error rate reflects an explicit choice of markers for which genotyping rates have been found to be highly repeatable and whose individual polymerase chain reactions (PCR) have been optimized. Non-differential genotyping errors, that is, those that do not differ systematically according to outcome status, will usually bias associations towards the null [65,66], just as for other non-differential errors. The most marked bias occurs when genotyping sensitivity is poor and genotype prevalence is high (>85%) or, as the corollary, when genotyping specificity is poor and genotype prevalence is low (<15%) [65]. When measurement of the environmental exposure has substantial error, genotyping errors of the order of 3% can lead to substantial under-estimation of the magnitude of an interaction effect [67]. When there are systematic differences in genotyping according to outcome status (differential error), bias in any direction may occur. Unblinded assessment may lead to differential misclassification. For genome-wide association studies of SNPs, differential misclassification between comparison groups (for example, cases and controls) can occur because of differences in DNA storage, collection or processing protocols, even when the genotyping itself meets the highest possible standards [68]. In this situation, using samples blinded to comparison group to determine the parameters for allele calling could still lead to differential misclassification. To minimize such differential misclassification, it would be necessary to calibrate the software separately for each group. This is one of the reasons for our recommendation to specify whether genotypes were assigned using all of the data from the study simultaneously or in smaller batches.

### Population Stratification

*Recommendation for reporting of methods (Table 1, item 12(h)): Describe any methods used to assess or address population stratification.*

Population stratification is the presence within a population of subgroups among which allele (or genotype; or haplotype) frequencies and disease risks differ. When the groups compared in the study differ in their proportions of the population subgroups, an association between the genotype and the disease being investigated may reflect the genotype being an indicator identifying a population subgroup rather than a causal variant. In this situation, population subgroup is a confounder because it is associated with both genotype frequency and disease risk. The potential implications of population stratification for the validity of genetic association studies have been debated [69–83]. Modelling the possible effect of population stratification (when no effort has been made to address it) suggests that the effect is likely to be small in most situations [75,76,78–80]. Meta-analyses of 43 gene-disease associations comprising 697 individual studies showed consistent associations across groups of different ethnic origin [80], and thus provide evidence against a large effect of population stratification, hidden or otherwise. However, as studies of association and interaction typically address moderate or small effects and hence require large sample sizes, a small bias arising from population stratification may be important [81]. Study design (case-family control studies) and statistical methods [84] have been proposed to address population stratification, but so far few studies have used these suggestions [49]. Most of the early genome-wide association studies used family-based designs or such methods as genomic control and principal components analysis [85,86] to control for stratification. These approaches are particularly appropriate for addressing bias when the identified genetic effects are very small (odds ratio <1.20), as has been the situation in many recent genome-wide association studies [85,87–105]. In view of the debate about the potential implications of population stratification for the validity of genetic association studies, we recommend transparent reporting of the methods used, or stating that none was used, to address this potential problem. This reporting will enable empirical evidence to accrue about the effects of population stratification and methods to address it.

### Modelling Haplotype Variation

*Recommendation for reporting of methods (Table 1, item 12(g)): Describe any methods used for inferring genotypes or haplotypes.*

A haplotype is a combination of specific alleles at neighbouring genes that tends to be inherited together. There has been considerable interest in modelling haplotype variation within candidate genes. Typically, the number of haplotypes observed within a gene is much smaller than the theoretical number of all possible haplotypes [106,107]. Motivation for utilizing haplotypes comes, in large part, from the fact that multiple SNPs may “tag” an untyped variant more effectively than a single typed variant. The subset of SNPs used in such an approach is called “haplotype tagging” SNPs. Implicitly, an aim of haplotype tagging is to reduce the number of SNPs that have to be genotyped, while maintaining statistical power to detect an association with the phenotype. Maps of human genetic variation are becoming more complete, and large scale genotypic analysis is becoming increasingly feasible. In consequence, it is possible that modelling haplotype variation will become more focussed on rare causal variants, because these may not be included in the genotyping platforms.

In most current, large-scale genetic association studies, data are collected as unphased multilocus genotypes (that is, which alleles are aligned together on particular segments of chromosome is unknown). It is common in such studies to use statistical methods to estimate haplotypes [108–111], and their accuracy and efficiency have been discussed [112–116]. Some methods attempt to make use of a concept called haplotype “blocks” [117,118], but the results of these methods are sensitive to the specific definitions of the “blocks” [119,120]. Reporting of the methods used to infer individual haplotypes and population haplotype frequencies, along with their associated uncertainties should enhance our understanding of the possible effects of different methods of modelling haplotype variation on study results as well as enabling comparison and syntheses of results from different studies.

Information on common patterns of genetic variation revealed by the International Haplotype Map (HapMap) Project [107] can be applied in the analysis of genome-wide association studies to infer genotypic variation at markers not typed directly in these studies [121,122]. Essentially, these methods perform haplotype-based tests but make use of information on variation in a set of reference samples (for example, HapMap) to guide the specific tests of association, collapsing a potentially large number of haplotypes into two classes (the allelic variation) at each marker. It is expected that these techniques will increase power in individual studies, and will aid in combining data across studies, and even across differing genotyping platforms. If imputation procedures have been used, it is useful to know the method, accuracy thresholds for acceptable imputation, how imputed genotypes were handled or weighted in the analysis, and whether any associations based on imputed genotypes were also verified on the basis of direct genotyping at a subsequent stage.

### Hardy-Weinberg Equilibrium

*Recommendation for reporting of methods (Table 1, item 12(f)): State whether Hardy-Weinberg equilibrium was considered and, if so, how.*

Hardy-Weinberg equilibrium has become widely accepted as an underlying model in population genetics after Hardy [123] and Weinberg [124] proposed the concept that genotype frequencies at a genetic locus are stable within one generation of random mating; the assumption of HWE is equivalent to the independence of two alleles at a locus. Views differ on whether testing for departure from HWE is a useful method to detect errors or peculiarities in the data set, and also the method of testing [125]. In particular, it has been suggested that deviation from HWE may be a sign of genotyping errors [126–128]. Testing for departure from HWE has a role in detecting gross errors of genotyping in large-scale genotyping projects such as identifying SNPs for which the clustering algorithms used to call genotypes have broken down [85,129]. However, the statistical power to detect less important errors of genotyping by testing for departure from HWE is low [130] and, in hypothetical data, the presence of HWE was generally not altered by the introduction of genotyping errors [131]. Furthermore, the assumptions underlying HWE, including random mating, lack of selection according to genotype, and absence of mutation or gene flow, are rarely met in human populations [132,133]. In five of 42 gene-disease associations assessed in meta-analyses of almost 600 studies, the results of studies that violated HWE significantly differed from results of studies that conformed to the model [134]. Moreover, the study suggested that exclusion of HWE-violating studies may result in loss of the statistical significance of some postulated gene-disease associations and that adjustment for the magnitude of deviation from the model may also have the same consequence for some other gene-disease associations. Given the differing views about the value of testing for departure from HWE and about the test methods, transparent reporting of whether such testing was done and, if so, the method used, is important for allowing the empirical evidence to accrue.

For massive-testing platforms, such as genome-wide association studies, it might be expected that many false-positive violations of HWE would occur if a lenient *P* value threshold were set. There is no consensus on the appropriate *P* value threshold for HWE-related quality control in this setting. So, we recommend that investigators state which threshold they have used, if any, to exclude specific polymorphisms from further consideration. For SNPs with low minor allele frequencies, substantially more significant results than expected by chance have been observed, and the distribution of alleles at these loci has often been found to show departure from HWE.

For genome-wide association studies, another approach that has been used to detect errors or peculiarities in the data set (due to population stratification, genotyping error, HWE deviations or other reasons) has been to construct quantile-quantile (Q/Q) plots whereby observed association statistics or calculated *P* values for each SNP are ranked in order from smallest to largest and plotted against the expected null distribution [129,130]. The shape of the curve can lend insight into whether or not systematic biases are present.

### Replication

*Recommendation: State if the study is the first report of a genetic association, a replication effort, or both. (Table 1, item 3)*

Articles that present and synthesize data from several studies in a single report are becoming more common. In particular, many genome-wide association analyses describe several different study populations, sometimes with different study designs and genotyping platforms, and in various stages of discovery and replication [129,130]. When data from several studies are presented in a single original report, each of the constituent studies and the composite results should be fully described. For example, a discussion of sample size and the reason for arriving at that size would include clear differentiation between the initial group (those that were typed with the full set of SNPs) and those that were included in the replication phase only (typed with a reduced set of SNPs) [129,130]. Describing the methods and results in sufficient detail would require substantial space in print, but options for publishing additional information on the study online make this possible.

## Discussion

The choices made for study design, conduct and data analysis potentially influence the magnitude and direction of results of genetic association studies. However, the empirical evidence on these effects is insufficient. Transparency of reporting is thus essential for developing a better evidence base (Table 2). Transparent reporting helps address gaps in empirical evidence [45], such as the effects of incomplete participation and genotyping errors. It will also help assess the impact of currently controversial issues such as population stratification, methods of inferring haplotypes, departure from HWE and multiple testing on effect estimates under different study conditions.

The STREGA Statement proposes a minimum checklist of items for reporting genetic association studies. The statement has several strengths. First, it is based on existing guidance on reporting observational studies (STROBE). Second, it was developed from discussions of an interdisciplinary group that included epidemiologists, geneticists, statisticians, journal editors, and graduate students, thus reflecting a broad collaborative approach in terminology accessible to scientists from diverse disciplines. Finally, it explicitly describes the rationale for the decisions (Table 2) and has a clear plan for dissemination and evaluation.

The STREGA recommendations are available at http://www.strega-statement.org/. We welcome comments, which will be used to refine future versions of the recommendations. We note that little is known about the most effective ways to apply reporting guidelines in practice, and that therefore it has been suggested that editors and authors collect, analyze, and report their experiences in using such guidelines [135]. We consider that the STREGA recommendations can be used by authors, peer reviewers and editors to improve the reporting of genetic association studies. We invite journals to endorse STREGA, for example by including STREGA and its Web address in their Instructions for Authors and by advising authors and peer reviewers to use the checklist as a guide. It has been suggested that reporting guidelines are most helpful if authors keep the general content of the guideline items in mind as they write their initial drafts, then refer to the details of individual items as they critically appraise what they have written during the revision process [135]. We emphasize that the STREGA reporting guidelines should not be used for screening submitted manuscripts to determine the quality or validity of the study being reported. Adherence to the recommendations may make some manuscripts longer, and this may be seen as a drawback in an era of limited space in a print journal. However, the ability to post information on the Web should alleviate this concern. The place in which supplementary information is presented can be decided by authors and editors of the individual journal.

We hope that the recommendations stimulate transparent and improved reporting of genetic association studies. In turn, better reporting of original studies would facilitate the synthesis of available research results and the further development of study methods in genetic epidemiology with the ultimate goal of improving the understanding of the role of genetic factors in the cause of diseases.

## Supporting Information

Table S1 STREGA Reporting Recommendations, Extended from STROBE Statement Editable version of Table 1.
(97 KB DOC).

## Abbreviations

HWE: Hardy-Weinberg equilibrium
SNP: single nucleotide polymorphism
STREGA: STrengthening the REporting of Genetic Association studies
STROBE: Strengthening the Reporting of Observational Studies in Epidemiology
